# Passing Positions of the Moment

**Published:** 1919-11-29

**Authors:** 


### Passing Positions of the Moment.

### Nursing Papers in Stormy Weather.

A MUSTARD poultice sometimes produces surprising results. An extraordinary incident is reported to have arisen at a meeting of the Standing Committee of the Central Midwives Board, held on November 12. The *British Journal of Nursing* sent a letter, "in consequence of the great rise in the cost of printing and paper, suggesting that the Board should pay a larger price for their advertisements in this publication." The Board decided for the time to pay 7s. 6d. per inch, instead of 5s., for its advertisements in the *B.J.N.* Possibly inspired by this adventure, a contemporary has promptly offered to send a copy of each issue for twelve weeks, postage free, on receipt of the sum of 1s. Whither are our contemporaries drifting? As the proverb has it, "One has always strength enough to bear the misfortunes of one's friends."

### In the Train.

"YES, it's all right coming over, we've just been living on the thoughts of 'leave' for months; but going back is another thing altogether. I'd forgot how beautiful England was till I came across from France this time!" "Beautiful?" ejaculated the other man, glancing through the window at the grey, damp haze. "Yes, sir, *beautiful* is what I said; houses whole, and with roofs on 'em; gardens and fields clean and tidy; no ruins about. Friendly folks around, and you able to walk anywhere and feel you're safe. Good Lord, *safe!* I'm blessed if folks at home know what that means. . . . For months I've seen desolate villages, ruined homesteads, trampled, blood-stained fields, and—well, sir, perhaps you'd rather not hear about the dead . . . they're always with *us*."

